# *Plasmodium chabaudi* malaria parasites adjust liver stage development to synchronise egressing blood stages with host daily rhythms

**DOI:** 10.1038/s42003-026-10641-x

**Published:** 2026-07-16

**Authors:** Alejandra Herbert-Mainero, Petra Schneider, Aidan J. O’Donnell, Sarah E. Reece

**Affiliations:** 1 Institute of Ecology and Evolution and Institute of Immunology and Infection, School of Biological Sciences, University of Edinburgh, Edinburgh, UK; 2 Department of Human Genetics, The University of Utah, Salt Lake City, UT USA

**Keywords:** Evolutionary ecology, Microbial ecology, Parasite host response, Malaria

## Abstract

Synchronised multiplication of *Plasmodium* parasites within red blood cells causes periodic malaria fevers. Aligning blood stage development with the feeding-fasting rhythm of the vertebrate host facilitates within-host survival and between-host transmission. We use the rodent model *Plasmodium chabaudi* to test when, following development in the liver, the blood stage of infection begins. We find egress from the liver into the blood is aligned with the time of day of rhythmic host feeding, but only in wild type hosts, with egress occurring after a fixed period of pre-erythrocytic development in hosts without a canonical circadian clock. However, perturbing the duration over which parasites enter the bloodstream does not affect their multiplication rate in the first five intraerythrocytic development cycles, suggesting that the expected fitness benefits from timing egress anticipates rhythmic challenges or opportunities (e.g. rhythmic nutrient limitation or nutrient availability) later in the infection when parasite densities increase.

## Abstract (web summary)

Malaria parasites adjust the time they egress from the liver into the blood according to when the host eats. This strategy requires time cues from the host circadian clock and rhythmic host feeding and enables parasites to exploit the host’s food.

## Introduction

The daily rotation of the Earth exposes organisms to predictable environmental cycles that impact fitness. Endogenous time-keeping mechanisms, including circadian clocks, are thought to have evolved to enable organisms to appropriately schedule activities to align with these environmental rhythms^1–3^. While the endogenous clocks of free-living organisms are the subject of much research, whether, how and why parasites keep time is poorly understood^4^. Parasites (including pathogens and microbes) are exposed to the daily rhythms expressed by hosts and vectors, including rhythms in immune responses, behaviours and nutrient availability that follow from hosts feeding during their active phase. Many parasites have complex life cycles, using multiple hosts/vectors, and must cope with physiological rhythms in their current host while also anticipating rhythms in the next host that dictate the time of day at which transmission occurs^5^. For example, malaria parasites (*Plasmodium spp.)* rhythmically invade and burst the red blood cells (RBCs) of their vertebrate host. This intraerythrocytic development cycle (IDC) lasts for multiples of 24 h, depending on the species. In the rodent malaria *P. chabaudi*, the IDC is timed to best exploit the nutrients that parasites need from the host’s food, while also ensuring that the production of transmission forms starts at the correct time of day to achieve full maturation by nighttime when mosquito vectors bite^5–13^. Alignment to host rhythms is actively maintained by a parasite circadian oscillator; when host feeding rhythms are experimentally shifted, *P. chabaudi* shortens the IDC by 2–3 h per cycle to regain alignment with host feeding rhythms^9–12,14–19^. Such plasticity comes with a cost to per-cycle productivity^9,12,20^, although completing additional rounds of multiplication during rescheduling can compensate for this cost^18,21^.

Like *Plasmodium*, many parasites reside in multiple tissues within each host/vector and therefore experience a variety of host tissue-specific rhythms during their life cycle, which provide time-of-day-specific opportunities to exploit or dangers to evade. Before the IDC begins, malaria parasites enter the vertebrate host during the bite of an infected mosquito and subsequently undergo extensive replication in hepatocytes. These liver-stage parasites multiply by several orders of magnitude more than blood stages and are intensely nutrient-demanding^22–25^. Because we expect that the circadian clock used during the IDC will also be ticking in liver-stage parasites, and because hepatocytes store nutrients rhythmically, temporal alignment to host feeding might benefit liver-stage parasites^10–12,14–19,26^. Alternatively, because replication of nuclei within liver-stage parasites occurs on a time scale much faster than 24 h^27–31^ and liver-stage parasites can alter hepatocyte transcription to secure resources^32–34^, this may relax any circadian constraint on nutrient acquisition by liver-stage parasites. In contrast, altering host cell transcription to maximise nutrient accessibility is not possible in mammalian RBCs (which lack a nucleus), forcing IDC stages to be more reliant on exploiting host feeding-fasting rhythms than liver-stage parasites. While karyokinesis is asynchronous in both blood and liver schizonts, the subsequent organellar and membrane biosynthesis in the multinucleated schizont as well as segregation of progeny by cytokinesis is fairly synchronous^27–31,35–41^. This opens avenues for liver-stage parasites to either synchronise cytokinesis or egress and start the IDC in alignment with host rhythms. Whether parasites schedule egress ‘on time’ to benefit the IDC, or whether liver-stage parasites are unable to synchronise cytokinesis or delay egress, thus forcing IDC stages to synchronise with each other and host rhythms once in the blood, are open questions.

Here, we ask whether *P. chabaudi* has evolved to start the blood stage of infection synchronously and aligned to host feeding-fasting rhythms by experimentally testing the following hypotheses. *Hypothesis* 1: parasites egress using time-of-day cues directly derived from host feeding-fasting rhythms as observed for IDC stages. For example, responding to isoleucine rhythms (which is a likely time of day cue for IDC stages^26^) would allow parasites to maximise fitness by ensuring the IDC is appropriately aligned to host feeding-fasting rhythms from the point of initiating the blood stage. *Hypothesis* 2: egress cues are derived from host TTFL-driven rhythms (the transcription-translation-feedback-loop that runs the canonical circadian clock, which is most responsive to light), and so indirectly reflecting the timing of host feeding-fasting rhythms. While this allows parasites to align egress with host feeding in general, natural perturbations to feeding schedules would cause misalignment, resulting in fitness costs. With considerable variation in the time of day at which infectious mosquito bites are received^42–45^, causing, for example, dawn and dusk bites to introduce parasites 12 h apart, both hypotheses 1 and 2 require the duration of liver stage development to be plastic. *Hypothesis* 3: parasites may have evolved a fixed duration of liver stage development, which matches the duration between the average time of day that infectious bites are received and the average time of day that hosts feed. This strategy ensures that egress usually coincides with host feeding-fasting rhythms and reduces the costs of detecting and interpreting time-of-day cues, but natural variation in mosquito biting time of day^42–45^ and/or the host’s feeding schedule would cause misalignment of IDC stages and fitness costs. *Hypothesis* 4: liver-stage parasites may be entirely unresponsive to host rhythms, completing development in an unsynchronised manner, and egress over a prolonged duration. This may be caused by differences in the rates of development or productivity (number of progeny produced) between individual liver-stage parasites within a host, and could be advantageous if, for example, gradual release into the blood avoids stimulating dangerous immune responses. We test these four hypotheses by manipulating when *P. chabaudi* infections are initiated by mosquito bite with respect to the phase of host feeding-fasting and TTFL-driven rhythms, using wild-type and TTFL-deficient mice. We also investigate the fitness consequences of a highly synchronous versus a temporally dispersed initiation of the blood stage of infection. We find that *P. chabaudi* parasites plastically adjust liver stage duration to align egress with host feeding, that this plasticity requires both the host canonical circadian clock and rhythmic host feeding cues, and we predict this may provide fitness benefits later in infections by mitigating resource limitation parasites may experience from being at high density.

## Results

Using an in vivo model system allowed us to manipulate the host’s feeding-fasting rhythms and/or TTFL-driven rhythms (i.e. the presence of a functional canonical circadian clock), relative to the time of day at which *P. chabaudi* (genotype AJ) infections are initiated, while also allowing parasites to respond to the full complexity of the daily rhythms and physiological processes they encounter inside a mammalian host. We carried out three experiments: the ‘Alignment of egress’ and ‘Time of egress’ experiments determine if the blood stage of infection is initiated in a rhythmic manner, when this occurs and in response to which host rhythms, and the ‘Simulation of egress’ experiment tests whether starting the IDC synchronously maximises blood stage multiplication rates.

### The influence of host rhythms on parasite egress from the liver

**Fig. 1:**
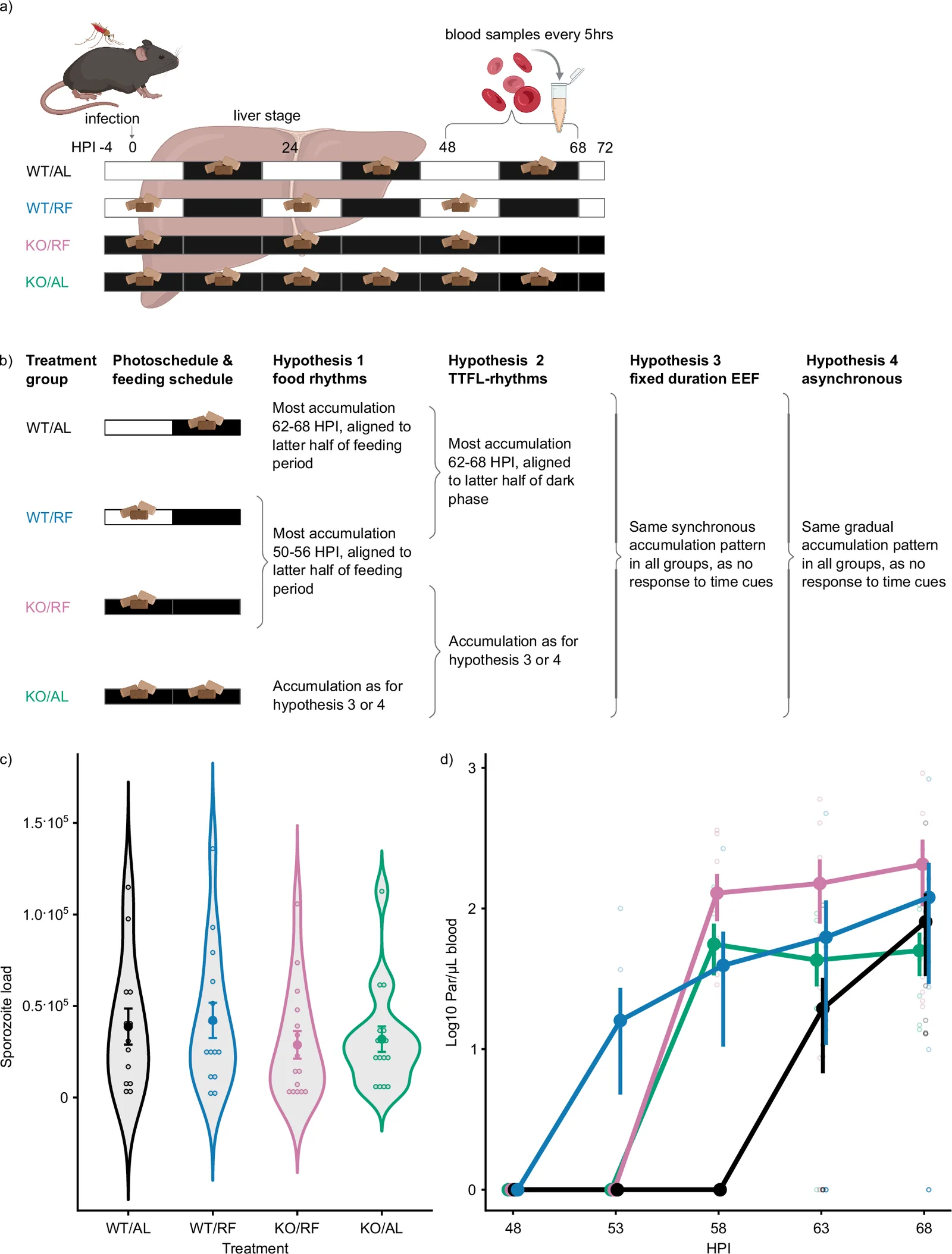
Alignment of egress experiment. **a** Experimental setup. *Plasmodium chabaudi* AJ liver stage infections were initiated via mosquito bite 4 h into the light phase (ZT4) for wild-type mice in a standard 12 h light (white):12 h dark (black) photoschedule (WT), and simultaneously in *Per1*^*-/-*^;* Per2*^*-/-*^ double KO mice in continuous darkness (KO) as denoted by the horizontal bars for each host type. For each host type, there were two treatment groups: one group was fed ad libitum (AL), and the other group’s feeding was restricted to a window of 12 h (RF). Mice experienced different feeding-fasting rhythms, with food pellets representing when mice eat based on either their natural nocturnal rhythms (WT/AL) or when food was available to them (WT/RF, KO/RF and KO/AL). **b** Treatment groups and predicted outcomes for the 4 hypotheses. The grouping of predicted patterns of parasite accumulation in the blood is shown for each of the 4 hypotheses. **c** Violin plot for sporozoite loads, approximated by residual sporozoites in the salivary glands, summed for all mosquitoes that fed on each exposed host. Datapoints show residual sporozoite loads per host, with mean ± sem shown for each treatment group. **d** Log_10_ mean *P. chabaudi* AJ densities per µL blood ± sem at 5-hourly intervals between 48 and 68 HPI for *n* = 8 infected (out of *n* = 15 exposed) WT/AL mice; 9 (15) WT/RF; 9 (16) KO/RF and 6 (16) KO/AL mice. HPI: hours post infection by mosquito bite; colours represent the different treatment groups. Figure 1a was created in BioRender. Schneider, P. (2026) https://BioRender.com/m94yuv5.

In the ‘Alignment of egress’ experiment, we asked whether the patterns of accumulation following the appearance of parasites in the blood are determined by either host feeding-fasting (hypothesis 1) or TTFL-driven (hypothesis 2) rhythms, or are unresponsive to host rhythms. Such unresponsiveness could occur either because the duration of liver stage development is fixed (hypothesis 3), or because egress occurs over a prolonged window (e.g. spanning most of a 24 h day, hypothesis 4). To distinguish between these four hypotheses, we initiated infections by mosquito bites in WT hosts under unperturbed conditions and in hosts whose TTFL-driven rhythms, feeding-fasting rhythms, or both were disrupted. We subsequently monitored the accumulation of blood stage parasites as a proxy for egress (Alignment of egress experiment, Fig. 1a). We manipulated the presence of TTFL-driven host rhythms by using either wildtype C57BL/6 mice in a standard 12 h light:12 h dark photoschedule (WT) or *Per1*^*-/-*^; *Per2*^*-/-*^ double KO TTFL-deficient mice^46^ (KO) that are arrhythmic when kept under constant conditions (constant darkness and ad libitum (AL) feeding)^47^. Specifically, WT hosts that are fed ad libitum (AL) eat in the dark phase, but KO mice in continuous darkness and fed ad libitum (AL) eat continuously. In addition to AL-fed groups for both WT and KO hosts, we experimentally controlled feeding schedules by restricting access to food to a 12-h window (RF) in both types of hosts. Restricted feeding for 12 h (RF) creates a feeding-fasting rhythm in the absence of TTFL-driven rhythms in KO mice and, when timed during the light phase, decouples the alignment between feeding-fasting and TTFL-driven rhythms in WT mice^12,48^.

We predicted that patterns of parasite accumulation in the blood across the 4 treatment groups (WT/AL, WT/RF, KO/AL and KO/RF) should vary between hypotheses 1 to 4 (Fig. 1b) as follows. Under hypothesis 1, we expect synchronous accumulation during the latter part of the feeding window for all groups with a feeding-fasting rhythm (WT/AL, WT/RF and KO/RF). Specifically, to align the IDC to host feeding, egress should occur towards the end of the dark/feeding period for the WT/AL group, with this pattern shifting by 12 h in both RF groups to match the hosts’ feeding windows. Under hypothesis 2, to align the IDC to host TTFL rhythms, both WT groups should follow identical, and synchronous, patterns of accumulation towards the end of the dark period, independent of host feeding-fasting schedules, whereas the KO groups should display the same pattern as each other, which is likely different from that of the WT groups. Under hypothesis 3, all groups should display the same and fairly synchronous pattern of accumulation due to a fixed duration of liver stage development. Whilst mosquito infections naturally occur at night, we infected all hosts during the light phase (according to the photoschedule of WT hosts) to ensure the expected time of egress under hypothesis 3 is decoupled from the dark/light cycle implicated by hypothesis 2. Finally, observing gradual accumulation over a prolonged window in all four groups would support hypothesis 4.

We initiated infections by exposing each mouse to the bites of 24 mosquitoes (54% *P. chabaudi* infection prevalence) at 11 am GMT, which is 4 h after lights on (zeitgeber time, ZT4) for the WT hosts (Fig. 1a and 'Methods'). As approximated by residual sporozoites, sporozoite exposure was consistent across treatment groups (*F*_3, 56_ = 0.558, *p* = 0.645, Fig. 1c). The relatively low sporozoite load of 35113 ± sem 4170 sporozoites per mouse resulted in 52% ± sem 6% of exposed hosts becoming infected, with equal prevalence between treatment groups (*χ*^2^(3) = 2.004, *p* = 0.572). Total parasite densities achieved at 68 HPI did not differ between treatment groups (*F*_3, 28_ = 2.072, *p* = 0.127), which—assuming the majority of parasites have egressed—suggests that host rhythms do not influence liver-stage parasite productivity. However, the dynamics of parasite accumulation varied between treatment groups (HPI by treatment interaction: *χ*^2^(9) = 29.87, *p* < 0.001; Fig. 1d), indicating variation in the timing of egress. This eliminates hypotheses 3 and 4 because they predict egress patterns to be the same across all treatment groups. Instead, the observed patterns support a response to host feeding-fasting (hypothesis 1) or TTFL-driven rhythms (hypothesis 2) (Fig. 1b). Hypothesis 1 is supported because parasites are first detected in the blood during the latter half of the feeding window for both WT groups (WT/AL, 63 HPI; WT/RF, 53 HPI) for which the feeding window was offset by 12 h. However, a role of TTFL-driven rhythms (hypothesis 2) is also supported because both KO/RF and KO/AL groups follow the same pattern of accumulation as each other (parasites first detected in the blood at 58 HPI), which differs from the patterns in the WT groups.

**Fig. 2:**
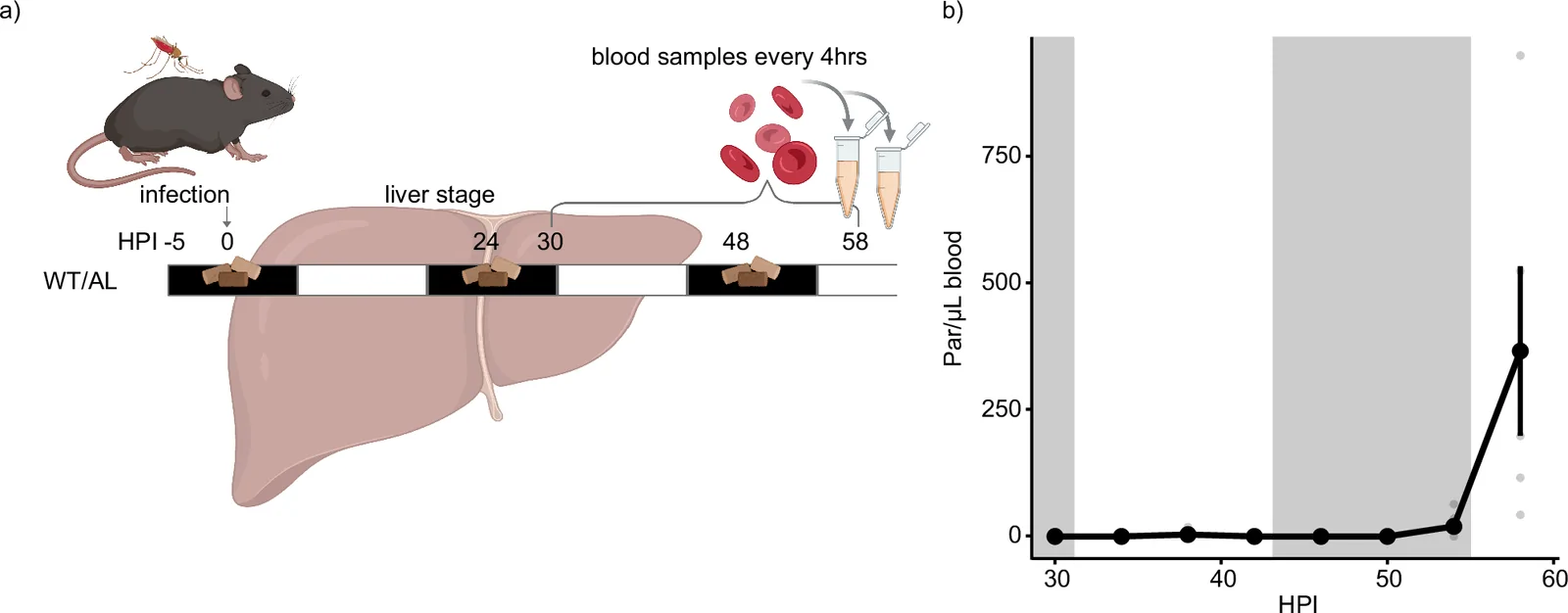
Time of egress experiment. **a** Experimental setup. *Plasmodium chabaudi* AJ liver stage infections were initiated via mosquito bite 5 h into the host dark phase (ZT17) for wild-type ad libitum fed mice in a standard 12 h light (white):12 h dark (black) photoschedule (WT/AL). Food pellets represent when mice were eating based on their natural nocturnal rhythms (WT/AL). **b** Mean *P. chabaudi* AJ densities per µL blood ± sem at 4-hourly intervals between 30 and 58 HPI for *n* = 5 infected (out of *n* = 13 exposed) WT/AL hosts infected by mosquito bite 5 h into the dark phase (ZT17). The shaded areas represent darkness during the 12 h light:12 h dark photoschedule. HPI: hours post infection by mosquito bite. Figure 2a was created in BioRender. Schneider, P. (2026) https://BioRender.com/zhb2xgs.

A combination of hypotheses 1 and 2 is plausible if the host’s canonical clock is necessary for parasites to egress in alignment with host feeding-fasting rhythms. This is consistent with parasites exhibiting a fixed duration of liver-stage development, which results in synchronous egress between 53 and 58 HPI (Fig. 1d) in the absence of host TTFL-driven rhythms, as observed in the KO groups. With *Anopheles* mosquitoes usually blood-feeding and consequently initiating infections during the mid-part of the night, egress after a fixed developmental duration of 53–58  HPI (revealed in the KO groups) would coincide with the end of the feeding period in normal circumstances (i.e. in WT/AL hosts). In this ‘Alignment of egress’ experiment, we deliberately shifted the point of infection by mosquito bite to the light phase for WT hosts to better distinguish between our hypotheses. Therefore, to test whether egress normally occurs at the end of the dark/feeding phase, we performed a second experiment (‘Time of egress’ experiment) where we infected WT/AL hosts by mosquito bite during the dark phase (Fig. 2a and 'Methods') and observed that parasites start accumulating in the blood at the end of the feeding window/dark phase (54 HPI, Fig. 2b). Because this is considerably earlier than in the WT/AL group in the ‘Alignment of egress’ experiment (63 HPI, Fig. 1d) it confirms that parasites in the ‘Alignment of egress’ experiment were plastically extending the duration of liver stage development to initiate the IDC in alignment with the host’s feeding-fasting rhythms.

### Fitness consequences of starting the first IDC synchronously

Having found support for parasites in normal circumstances (i.e. WT/AL hosts) setting their egress schedule to align with the timing of host rhythms, we tested the fitness consequences of this strategy with a ‘Simulated egress’ experiment. We predicted that egress over a defined window towards the end of the feeding/dark phase is beneficial for parasite fitness because it allows IDC stages to garner the nutrients needed from the host’s feeding ('extrinsic fitness benefits'^9,18^), but there may also be 'intrinsic fitness benefits' specifically from synchronous development. For example, temporally compartmentalising DNA replication and metabolism is an assumed intrinsic benefit of using circadian clocks to schedule these processes across the tree of life^1–3^.

**Fig. 3:**
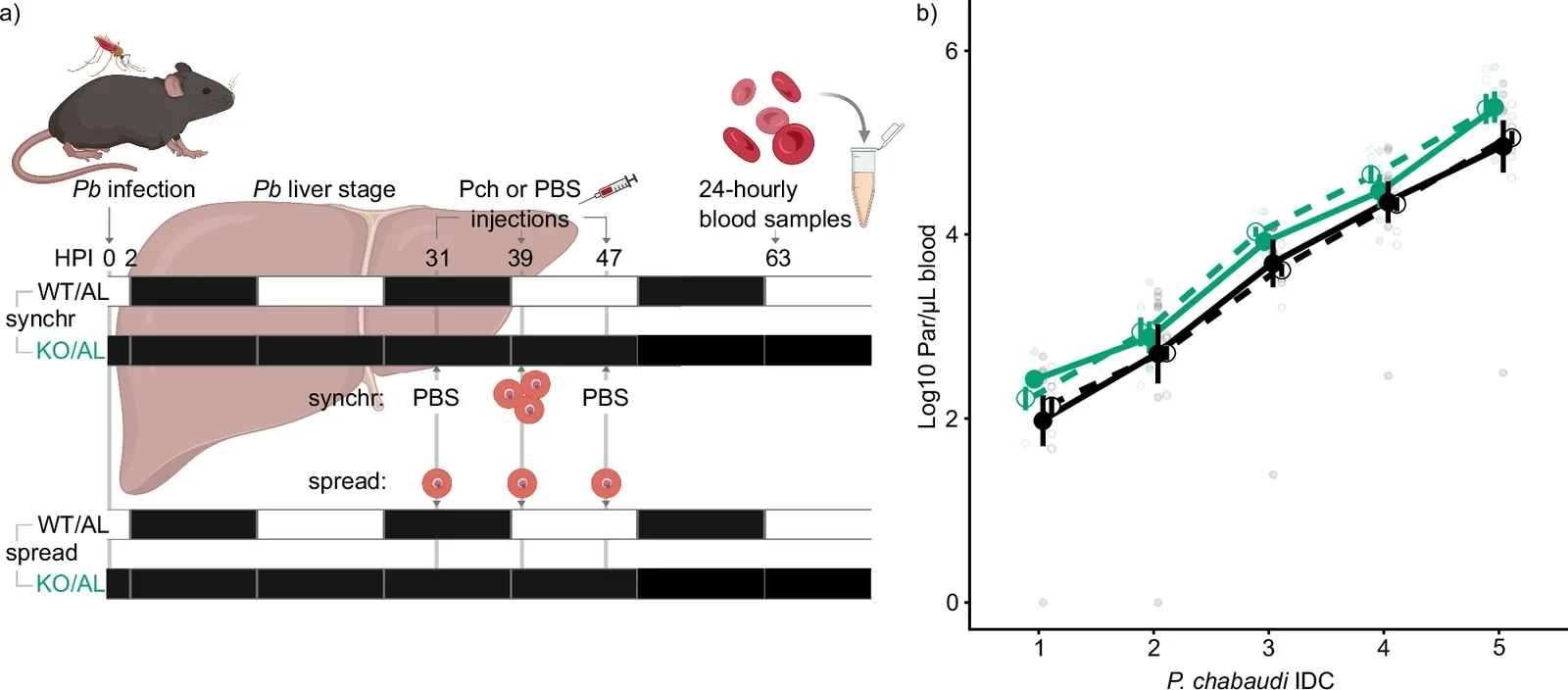
Simulated egress experiment. **a** Experimental design testing for fitness consequences of synchronous (synchr) or spread-out (spread) establishment of the IDC. Mice were exposed to *P. berghei* (Pb) infected mosquitoes 10 h into the host light phase (ZT10) for wildtype mice in a standard 12 h light (white):12 h dark (black) photoschedule (WT, *n* = 10 per group), and simultaneously in *Per1*^*-/-*^;* Per2*^*-/-*^ double KO mice in continuous darkness (KO, *n* = 5 per group) as denoted by the horizontal bars for each host type. Subsequently, all mice received 3 × 10^5^ *P. chabaudi* (Pch) ring-stage infected RBCs by injection. Inoculation regimes are 'spread' (10^5^ parasites each at 31, 39 and 47 HPI) or 'synchr' (3 × 10^5^ parasites at 39 HPI and PBS placebo at 31 and 47 HPI). **b** Log_10_ mean *P. chabaudi* AJ densities per µL blood ± sem for spread-out (dashed) and synchronous (solid) simulated egress strategies from 63 to 159 h after exposure to *P. berghei* infected mosquitoes (*P. chabaudi* IDC 1–5), in WT (black) and KO (green) hosts. HPI: hours post-infection by mosquito bite. Figure 3a was created in BioRender. Schneider, P. (2026) https://BioRender.com/a6n7ogb.

To test whether the pattern of egress affects the multiplication rate of IDC stages, we experimentally simulated 'synchronous' versus 'spread-out' egress patterns. Infections were either started with a single inoculum of IDC parasites at ring stage aligned to host feeding rhythms, or with three injections of equal numbers of ring stages spaced 8 h apart in which only one inoculum was aligned to host feeding rhythms (Fig. 3a). Furthermore, we also tested whether the consequences of starting the IDC in a synchronous or spread-out manner depend on whether hosts have a functional (WT/AL hosts) or disrupted (KO/AL hosts) TTFL clock. We predicted that if parasites in WT hosts multiply faster when egress is synchronous compared to spread out, they garner extrinsic benefits and that if this difference occurs in KO hosts, this indicates intrinsic benefits also occur.

We infected WT (in a 12 h light:12 h dark photoschedule), and KO (in constant darkness) hosts with *P. berghei* by mosquito bite to initiate a liver stage infection before receiving *P. chabaudi* blood stage parasites *via* injection (Fig. 3a and 'Methods'). We pre-infected hosts to maximise ecological relevance, such that the injected *P. chabaudi* parasites establish the blood-stage infection in hosts experiencing a liver-stage infection. Quantifying *P. chabaudi* using a species-specific quantitative PCR that does not detect *P. berghei* parasites prevented any liver-egressed parasites from confounding our results^49^. Each mouse was exposed to on average, 4.0 ± sem 0.2 *P. berghei*-infected mosquitoes, with no differences between treatment groups (*F*_3,26_ = 0.592, *p* = 0.626). From 31 to 47 h after *P. berghei* infections were initiated, each host received three injections, spaced 8 h apart (Fig. 3a). To simulate synchronous egress, *P. chabaudi* ring stages were administered only in the middle injection, timed to align the IDC to host rhythms for the WT hosts (a few hours into the egress window, i.e. 1 h after lights on, ZT1), and the same number of ring stage parasites were split equally across all three injections to simulate spread-out egress (Fig. 3a and 'Methods').

Over the 5 days following *P. chabaudi* infection, neither densities (*χ*^2^ (3) = 2.348, *p* = 0.504) nor multiplication rates (5.9± sem 0.3-fold growth/day) of parasites differed between treatment groups (day by treatment interaction: *χ*^2^(12) = 7.454, *p* = 0.826; Fig. 3b). Thus, starting the IDC synchronously or spread over 16 h does not impact parasites’ ability to establish a blood stage infection in rhythmic or arrhythmic hosts, suggesting that parasites do not receive extrinsic or intrinsic benefits from rhythmic egress, at least during the first IDCs.

## Discussion

We conducted two experiments to test whether *P. chabaudi* egresses from the liver and starts the IDC in alignment with the host’s feeding-fasting rhythms to optimise the development and multiplication of blood-stage parasites. We tested four hypotheses and do not find clear, unequivocal support for any of them individually. However, our results are consistent with a scenario in which parasites can adjust the duration of liver-stage development to align egress with host feeding-fasting rhythms, but require a time cue from the host’s canonical (TTFL) circadian clock to align with the timing of feeding. This is a more parsimonious explanation than disrupting the alignment between rhythms driven by the light-dark cycle and by feeding-fasting causing parasites in the WT/RF group to egress at a different time to those in the WT/AL group. If such internal desynchrony of host rhythms was the cause, parasites in KO/RF hosts would also have initiated the blood stage at the end of the feeding window because feeding-fasting provides the dominant time-of-day information in KO hosts (in constant darkness). Our third experiment probed whether highly synchronous egress, timed to align with the host’s feeding-fasting rhythms, enhances the multiplication rate of IDC stages. Contrary to expectations, the multiplication rate during the first five IDCs was similar for synchronous or temporally spread out (over 16 h) initiation of the blood stage.

Studies that determine the developmental duration from the liver to the blood stage of infection are sparse, especially for *P. chabaudi*. Our data indicate that *P. chabaudi* liver-stage parasites typically egress 53–58 HPI, consistent with previous estimates of 52–53 h^50^, and we also demonstrate, for the first time, that this duration can be plastically adjusted to align egress with host feeding. Altering the time of infection by mosquito bite for unperturbed WT hosts between the ‘Alignment’ and ‘Time of egress’ experiments confirms that egress starts at the end of the host feeding phase, matching the schedule of the IDC in established infections (Figs. 1d and 2). In addition, following a feeding window shift of 12 h, parasites of the WT/RF group egressed ~10 h earlier compared to the WT/AL fed control group, which is as close to 12 h as our 5-hourly sampling schedule allows (Fig. 1d). Plasticity in the duration of liver stage development may be expected given the variable duration of liver stage development observed across the *Plasmodium* genus. Compared to rodent malaria parasites, the human-infecting malaria parasites require much longer liver stage development (e.g. 6–10 days for *P. falciparum, P. ovale, P. knowlesi* and *P. vivax*, and up to 15 days for *P. malariae*) and some species produce dormant hypnozoites that remain in the liver for years before egressing into the blood. Relapse of *P. vivax* hypnozoites can occur in response to fever-inducing parasitic and bacterial (but not viral) infections, suggesting that reactivation is responsive to the host’s inflammatory response and thus may be influenced by circadian clocks^51,52^. Furthermore, the development of liver-stage parasites mirrors that of IDC stages, with a growth phase followed by multiple, asynchronous rounds of DNA replication and karyokinesis, and finally a fairly synchronous mass cytokinesis event that precedes egress from the host cell schizonts^27–31,35–41^. The duration of the IDC and the number of progeny per schizont varies between species and in response to environmental conditions. For example, culturing conditions (RBCs, nutrients) affect the duration of the IDC for *P. falciparum* and *P. knowlesi*^30,41,53^, and *P. chabaudi* re-aligns to host feeding-fasting rhythms by shortening the IDC, potentially by responding to blood isoleucine concentration^18,26^. These data show that the duration of both IDC and liver-stage development is species-dependent, whilst parasites across multiple *Plasmodium* species maintain plasticity to adapt this duration to fluctuating environmental conditions.

The signals used by parasites to end the liver stage and begin the blood stage of infection are unknown^29^. Parasites could use time-of-day cues sensed at the point of infection (or after) to set the schedule for the development of liver-stage parasites, or liver-stage parasites could complete development and wait for a time-of-day signal (a 'gate') to egress, or use a combination of both strategies. The different accumulation patterns in the WT and KO/RF groups suggests that the cues for egressing ‘on time’ involve the canonical (TTFL) circadian host clock. This was unexpected because IDC stages only need cues from the host’s feeding-fasting rhythm (potentially, blood isoleucine concentration^26^) to set their schedule. Fundamental differences between liver-stage parasites and IDC stages in nutrient acquisition and host-cell environment may explain liver-stage parasites’ dual response to food intake and the TTFL. Liver-stage parasites have no haemoglobin to digest, are highly dependent on lipid synthesis, and do not appear to have the new permeability pathways (NPPs) that facilitate the increased isoleucine uptake by infected erythrocytes^34,53,54^. Furthermore, rhythmic cues may differ in level and amplitude between the hepatocytes and RBCs, and liver-stage parasites may require higher-amplitude rhythms compared to IDC stages due to their nutrient-rich environment. Combining a TTFL- and food-driven response may result in more reliable egress timing when the host eats at an unusual time of the day, or for hosts under energetic stress that have an altered feeding-fasting rhythm. Thus, liver-stage parasite egress may be governed by both TTFL and feeding cues, analogous to the dual cue regulation proposed for the conversion of *Plasmodium* IDC stages into sexual transmission stages^55,56^. More generally, responding to dual cues facilitates the perception of noise^56^ and provides a more robust response in variable environments. Furthermore, the dampening of feeding-imposed liver rhythms in TTFL-deficient hosts^57^ could also explain why egress was not aligned to the timing of feeding in KO/RF hosts. For example, the rhythmicity of many lipid species, glucose, glycogen and triglycerides requires the presence of either the central TTFL clock or the peripheral liver TTFL clock^26,57–62^ and hormones like insulin and glucocorticoids act as systemic entrainer of hepatic rhythms to feeding^63–65^. Daily oscillations in hepatocyte translation efficiency, protein accumulation and cell size—processes that peak at the start of the fasting phase (light phase for mice) but depend on the alignment of photoschedule and feeding rhythms^66^—may further erode liver rhythms in KO hosts, preventing parasites from time-keeping. These oscillations are absent in mature erythrocytes, and so a reliance on the TTFL-driven aspects would be a constraint in IDC stages, perhaps explaining their lack of TTFL-dependency in IDC rhythmicity.

We also investigated the fitness benefits for parasites of adopting a plastic strategy for timing the initiation of the blood stage of infection. The evolution of adaptive plasticity requires a predictably fluctuating environment which can be reliably assessed, such as a repeatable environmental rhythm. Additionally, the energetic costs associated with sensing and responding to environmental cues must be outweighed by the fitness benefits of adopting a context-specific phenotype. Contrary to our expectations that a tightly synchronised egress, aligned to host feeding-fasting rhythms, would be favoured, a variable duration of the liver stage did not alter liver stage parasite productivity (Alignment of egress experiment, Fig. 1d). Although low parasite densities may obscure subtle group differences, our multi-IDC ‘Simulated egress’ experiment also showed that a synchronous start conferred no advantage to parasites in WT or KO hosts. This agrees with predictions that (IDC) schizonts burst when maximum size/progeny number is reached rather than after a specific duration of development^30,31,39^. Applying this strategy to liver-stage parasites suggests they may not gain a fitness advantage through increased productivity, but that the timing/synchrony of egress could be adaptive. Putative benefits of starting the blood-stage infection at a specific time of day could include overwhelming immune defences, evading peak immune activity, or minimising nutrient limitation. These factors may be negligible during the first IDCs when parasite densities remain low, but setting the right IDC schedule early ensures parasites are ‘on time’ when immune responses and nutrient limitation develop as infections progress, in response to rapidly increasing parasite densities. Repeating the ’Simulated egress’ experiment by starting infections with a higher parasite density in semi-immune or nutrient limited hosts is necessary to test whether the fitness benefits of egressing at the end of the host’s feeding window become apparent when infections progress. Furthermore, our approach for simulating spread-out egress may not have resulted in enough parasites being out of alignment with host rhythms to detect fitness costs. Parasite densities at the start of the infections were low, and while parasites in the first and third inocula were misaligned by 8 h, this may not be enough to significantly reduce replication^18,21^. For example, egress by *P. yoelii* and *P. berghei* liver-stage parasites spans 6–10 h, similar to our observations in *P. chabaudi*^24,67^. Such intermediate levels of synchrony could diffuse nutritional demand over a broader interval, thus moderating competition between genetically related parasites within the host^68,69^. Our fully synchronous and spread-out treatments may therefore be equidistant from the true optimum of intermediate synchrony, obscuring fitness differences. Thus, the best strategy may be for parasites to use host time-of-day cues to plastically adjust the duration of liver-stage parasite development but allow enough cell-cell variation in sensitivity to cues to ensure egress is not too synchronous.

Observations that nuclear division within liver-stage parasites is arrhythmic^27,28^, combined with our finding that liver egress is plastic and aligned with host feeding-fasting rhythms in normal hosts, indicate that rhythmicity is established between cytokinesis in liver-stage parasites and the first IDC. That this plastic scheduling of egress fails in TTFL-deficient hosts (Fig. 1d) is unlikely to matter in natural conditions where circadian clocks are omnipresent, but understanding how parasite rhythms interact with host rhythms is instrumental to elucidate the underlying mechanisms. Recognising that the liver stage can establish the rhythmic development of *Plasmodium* parasites in the blood has several implications. It provides a framework for interpreting how disturbed daily rhythms in humans and mosquito vectors might modulate malaria transmission. For example, plasticity in the time of day that parasites egress into the blood would considerably mitigate any negative impacts that the ongoing shift in *Anopheles* blood feeding times^42–45^ may have on parasites, compared to blood feeding at night^70^. Elucidating the signalling pathways underlying plasticity in liver-stage parasite maturation could lead to interventions that curb infections at a pre-symptomatic stage. Because the transition from asynchronous karyokinesis to synchronous and timed cytokinesis/egress occurs in liver-stage parasites and IDC schizonts^27–31,35–41^, agents that disrupt parasite time-keeping have the potential to impair multiple *Plasmodium* life stages simultaneously.

## Methods

**Fig. 4:**
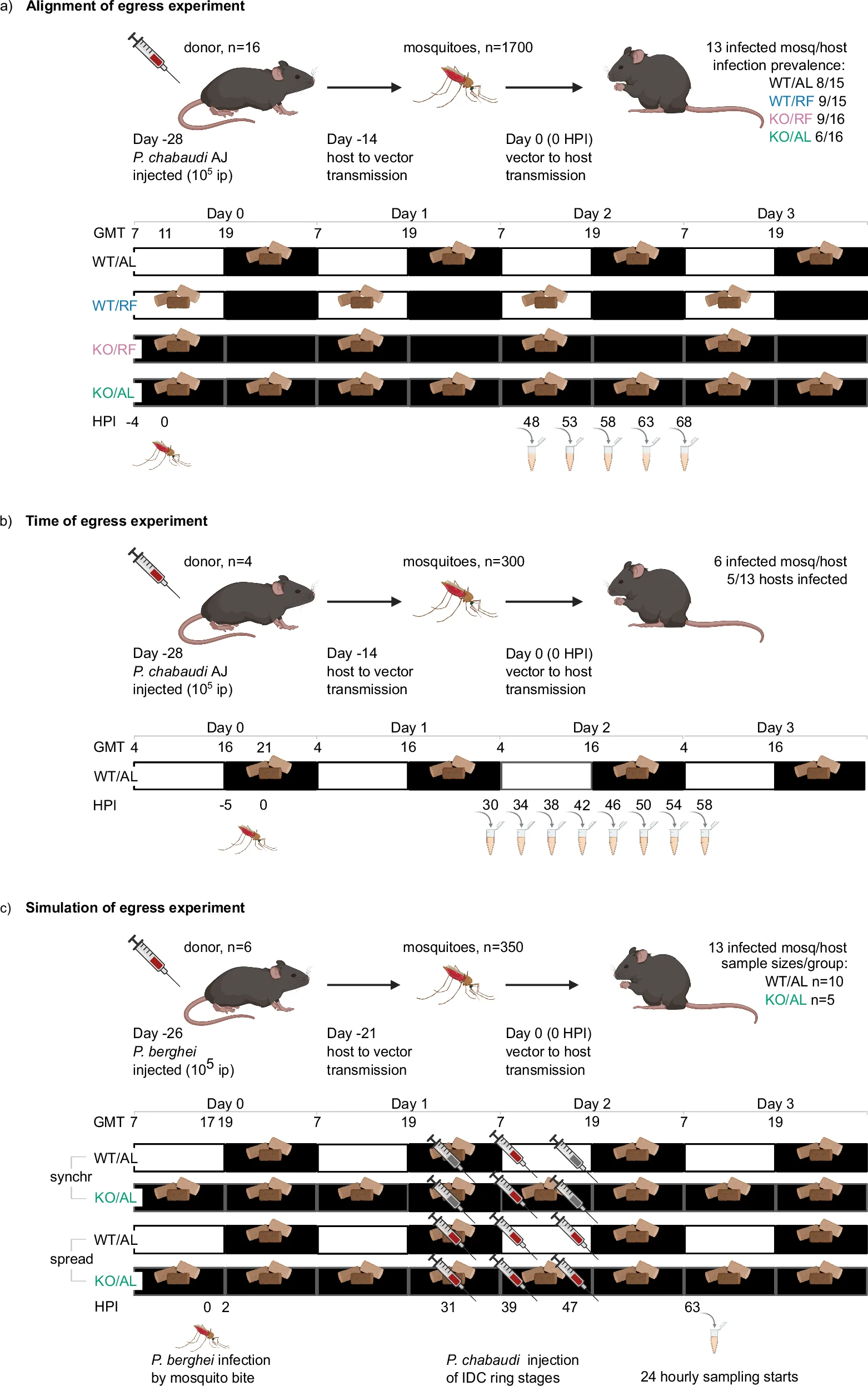
Experimental designs. **a** Alignment of egress experiment. **b** Time of egress experiment. **c** Simulated egress experiment. The top section of each graph depicts the timeline for infection of donor mice, infection of mosquitoes and subsequent infection of mice by mosquito bite, with experimental groups and sample sizes shown. The lower section of each graph illustrates the photoschedules (white: 12 h light; black: 12 h darkness) and feeding-fasting schedules (pellets: feeding window) for all treatment groups. Sampling time points are shown by illustration of tubes, and injection needles depict the intraperitoneal (ip) injection of *Plasmodium parasites* (red) or PBS (grey). WT: wildtype mice; KO: Per1^-/-^; Per2^-/-^ double KO mice; AL: ad libitum fed hosts, eat in darkness for WT and continuously for KO mice in continuous darkness; RF: restricted feeding for 12 h, creates a feeding-fasting rhythm in the *Per1*^*-/-*^*; Per2*^*-/-*^ double KO mice in continuous darkness^12,48^; HPI: hours post infection by mosquito bite; GMT: Greenwich mean time. Fig. 3a was created in BioRender. Schneider, P. (2026) https://BioRender.com/0l88zv7.

We performed three experiments: the ‘Alignment of egress’ and ‘Time of egress’ experiments tested whether parasites egress in line with host rhythms, and the ‘Simulated egress’ experiment quantified the fitness consequences of synchronous versus temporally dispersed egress from the liver. Treatment groups are described in the results section, with detailed experimental designs and procedure timelines in Fig. 4.

### Parasites, hosts and vectors

We used *P. chabaudi chabaudi* genotype AJ for liver and blood-stage infections in the **‘**Alignment of egress’ and ‘Time of egress’ experiments and for the blood stage in the ‘Simulated egress’ experiment. We used *P. berghei* ANKA for liver-stage infections in the ‘Simulated egress’ experiment because, due to its preference for young RBCs^71^, *P. berghei* is unlikely to interfere with the replication of *P. chabaudi* IDC stages, and *P. chabaudi* parasites can be separately quantified using quantitative PCR (qPCR,^49,72^).

To independently manipulate the hosts’ feeding-fasting rhythms and the rhythms generated by their canonical circadian clock (TTFL-driven rhythms), we used either wild-type (WT) C57BL/6 or *Per1*^*-/-*^; *Per2*^*-/-*^ double KO^46^ (KO) mice that are arrhythmic when housed in constant conditions (constant darkness, ad libitum feeding)^47^. All mice were mixed sexes, 8–10 weeks old, housed at 21 °C, and fed standard RM3 pelleted diet (801700, SDS, UK) with unrestricted access to drinking water supplemented with 0.05% para-aminobenzoic acid^73^. Mosquito vectors were *Anopheles stephensi*, reared at 26 °C, 70% relative humidity, in a 12 h light: 12 h dark photoschedule, with ad libitum access to 8% fructose post emergence.

### Ethics

We have complied with all relevant ethical regulations for animal use. Experimental procedures complied with all relevant ethical regulations for animal use, specifically the UK Animals Scientific Procedures Act 1986 (SI 2012/3039; PPL PP8390310). The study was conducted in accordance with the local legislation and institutional requirements and was approved by the Animal Welfare and Ethical Review Body at the University of Edinburgh.

### Transmission of parasites to mosquitoes

Donor mice received 10^5^ RBCs parasitised with *P. chabaudi* AJ or *P. berghei* intraperitoneally. In addition, *P. berghei* donor mice were treated with 125 mg kg^−1^ phenylhydrazine 3 days prior to *P. berghei* inoculation to stimulate anaemia and subsequent gametocytogenesis. Once gametocytes were observed by microscopic examination of Giemsa-stained thin blood smears, on day 14 (*P. chabaudi*) or day 5 (*P. berghei*) post inoculation, female *Anopheles stephensi* mosquitoes (3–5 days post emergence) fed for 15 min on groups of anaesthetised donors (500 µg kg^−1^ medetomidine, 50 mg kg^−1^ ketamine hydrochloride, intraperitoneally), following Spence et al.^74^. After the feed, euthanasia of anaesthetised mice was performed by cervical dislocation and confirmed by observation of rigor mortis (approved schedule 1/humane killing methods). Sample sizes for the ‘Alignment of egress’ and ‘Time of egress’ (*P. chabaudi*) and the ‘Simulated egress’ (*P. berghei*) experiments were 16, 4 and 6 gametocyte donor mice and 1700, 300 and 350 exposed mosquitoes, respectively (Fig. 4). Exposed mosquitoes were housed at 26 °C (*P. chabaudi*) or 21 °C (*P. berghei*). We quantified the prevalence of infected mosquitoes from oocyst counts obtained from 0.05% mercurochrome-stained midguts, dissected on day 8 (*P. chabaudi*, ‘Alignment of egress’ *n* = 206/1700, ‘Time of egress’ *n* = 20/300) or day 13–14 (*P. berghei*, ‘Simulated egress’ *n* = 50/350) post infection by mosquito bite (dPI). At 13 (*P. chabaudi*) or 20 (*P. berghei*) dPI, we randomly allocated mosquitoes to 62, 13 or 13 pots, containing 24, 20 or 8 mosquitoes each, of which on average 13.0, 6.2 or 6.2 mosquitoes were infected with *Plasmodium* (based on oocyst prevalence) for the ‘Alignment of egress’, ‘Time of egress’ and ‘Simulated egress’ experiments, respectively.

### Generating liver-stage and blood-stage infections

Liver infections were generated by exposing anaesthetised (500 µg kg^−1^ medetomidine, 50 mg kg^−1^ ketamine hydrochloride, intraperitoneally) hosts to one pot of mosquitoes each for 20  min^74^, after which anaesthesia was reversed using 2.5 mg kg^−1^ of atipamezole subcutaneously. Residual *P. chabaudi* sporozoite loads in the salivary glands of all mosquitoes feeding on an individual host were quantified by qPCR^8,75^ at the day of transmission to confirm that estimated sporozoite exposure was not different between treatment groups.

Blood stage infections in the ‘Simulated egress’ experiment were established by intraperitoneal injection of 3 × 10^5^*P. chabaudi* AJ ring-stage-infected RBCs at 39 HPI (ZT 1, i.e. 1 h after lights go on) and additional injections of PBS at 31 (ZT 17) and 47 HPI (ZT9), or by three doses of 10^5^ each at 31, 39 and 47 HPI^74^. These time points were chosen so that simulated egress of ring stages at 39 HPI (ZT1) occurs in line with host rhythms, i.e. the injected ring stages originate from *P. chabaudi* AJ-infected donor hosts that were kept at the same photoschedule as the recipient hosts. Ring stage parasites injected at the 31 and 47 HPI time points originated from *P. chabaudi* AJ-infected donor hosts that were kept at photoschedules that were advanced or delayed by 8 h. Therefore, the ring stage parasites in the simulated 'spread' group cover a window of 16 h (out of a 24-h IDC).

### Perturbation of rhythms and sample collection

Feeding-fasting rhythms were perturbed by restricting food (RF) availability to 12 h to shift the feeding window in WT mice or create a feeding-fasting rhythm in the KO mice in DD^12,48^. All mice were acclimated to their photoschedules and feeding-fasting regimes for at least two weeks prior to *Plasmodium* infections. Restricted feeding does not result in weight loss^18^, which we confirmed in the ‘Alignment of egress’ experiment by comparing weights between ad libitum (AL) fed and RF groups before infection (*F*_1, 29_ = 2.00, *p* = 0.168). During all experiments, blood was collected from the tail vein to quantify RBC densities by Coulter counter (2 µL, Beckman Coulter) and parasites (10 µL for qPCR^49,72^). At the end of the experiments, euthanasia was performed with a lethal dose of pentobarbital followed by cervical dislocation (approved schedule 1/humane killing methods).

### Molecular protocols

DNA was extracted from 10 µL blood using a semi-automatic Kingfisher Flex Magnetic Particle Processor and MagMAX^TM^-96 DNA MultiSample Kit (Thermo Fisher Scientific), following^76^, except for doubling the sample volume. *P. chabaudi* IDC stages were quantified by qPCR targeting the 5′ region of the PSOP1 gene (PCHAS_0620900)^72^, and an AJ-specific section of the 3′ region of the same gene in the ‘Simulated egress’ experiment^49^. Primers (900 nM) and probe (250 nM) for the PSOP1 5′ assay^72^ were: FW 5′-CAC AAT ATA GTA TAA AAG TAG GAC TTG AAA ATA ATA GTA G-3′, RV 5′-GGA ATA TGG GAT ATT GTC AAA GGA TAT AC-3′, probe: 5′-6FAM-TTT TCC ACT TAC AAC TCC A-MGB-3′. Primers (900 nM) and probe (250 nM) for the AJ-specific PSOP1 3′ assay^49^ were: FW: 5′-CCG AAT CAT TAT GTT TTA CTA TAA TGG GAA TA-3′; RV: 5′-AAA ATT GCA TAT ATT CCT AAA GTA CTA TCT ACT TCA TTA T-3′, probe: 5′-6FAM-CAA AGT AGA CAA TGC ACC AAT-MGB-3′. Both PSOP1 assays were run at 95 °C 20 s, followed by 40 cycles of: 95 °C 1 s, 60 °C 20 s. Quantification was relative to a serial dilution of microscopy-counted *P. chabaudi* AJ IDC stages, run in duplicate alongside our experimental biological replicates, with a correlation of *r*^2^ > 0.99 between parasite densities and PCR quantification cycles for all experiments and a coefficient of variation between technical replicates of <1.5%. PCR efficiency did not differ across plates for the ‘Alignment of egress’ (93 ± 2%, *F*_4, 51_ = 0.16, *p* = 0.96), ‘Time of egress’ (95 ± 2%, *F*_3, 36_ = 1.42, *p* = 0.25) or ‘Simulated egress’ (99 ± 2%, *F*_3, 48_ = 1.66, *p* = 0.19) experiments. No amplification was detected in negative (water) controls, and variation between technical replicates of our standards were negligible down to a density of 5 parasites µL^−1^ of blood, to which we set the lower limit of quantification.

Residual sporozoites in the salivary glands were quantified as described in Schneider et al.^8^. In brief, blood-fed mosquitoes were bisected^77^, and the head-thorax portions were pooled in groups of 3–6 from within the same pots to extract DNA^8^. Non-exposed mosquitoes were processed as negative controls. Sporozoites were quantified by qPCR targeting the 18S rRNA gene^75^, using a serial dilution series of *P. chabaudi* DNA from IDC ring stages that was run in duplicate alongside our experimental biological replicate samples. As both IDC ring stages and sporozoites are haploid, this allows for relative quantification of parasite numbers based on genome copies. The 18S qPCR to quantify sporozoites was run at 95 °C 2 min, followed by 40 cycles of 95 °C 3 s and 60 °C 30 s, using FW primer (300 nM) 5′-TGT CAG AGG TGA AAT TCT TAG ATT TTC T-3′, RV primer (300 nM) 5′-ACT TTC GTT CTT GAT TAA TGG AAG TAT TT-3′, and probe (200 nM) 5′-6FAM-CAA ACA ACT GCG AAA GC-MGB 3′. Amplification efficiency was 93 ± 2.7%, with a correlation between parasite genomes and PCR quantification cycles (*r*^2^) of 0.99. We approximated sporozoite exposure by summing sporozoite counts for all pools of fed mosquitoes for each mouse.

### Statistics and reproducibility

Data analysis was performed using R version 4.5.0 (2025-04-11). We used linear models to compare PCR efficiencies across plates and to compare mouse weights and sporozoite exposure (square-root transformed to meet model assumptions of normality and homogeneity of variance) between treatment groups. Infection prevalence was compared between treatment groups using generalised linear models with a binomial distribution. To compare how log_10_ parasite densities changed over time (HPI or dPI, treated as a factor to allow for non-linearity) between treatment groups, we constructed generalised linear mixed-effects models, testing for the interaction between HPI or dPI and treatment group, including the underlying fixed effects with mouse identity fitted as a random effect to account for repeated measures ('glmmTMB' R package). Generalised linear mixed-effects models used a Gaussian error distribution, with added zero-inflation in the ‘Alignment of egress’ experiment, where earlier time points were more likely to be negative. Only mice confirmed to be infected during at least one time point were included. For all analyses, model assumptions were tested using 'Dharma' package simulations. Model selection was performed via model fit (AICc) or log-likelihood ratio test (LRT) for nested models. Test statistics and associated *p*-values, with effect sizes for significant results, are presented in the main text. All figures represent the mean ± sem of data.

### Reporting summary

Further information on research design is available in the Nature Portfolio Reporting Summary linked to this article.

## Supplementary information

Reporting Summary

## Data Availability

The datasets supporting the conclusions of this article are available in the Edinburgh DataShare repository: https://doi.org/10.7488/ds/8049. The rodent malaria parasites *P. chabaudi* AJ and *P. berghei* ANKA can be obtained from the European Malaria Reagent Repository (http:// www.malariaresearch.eu).
