# Supplementary material for: Plasmodium chabaudi malaria parasites adjust liver stage development to synchronise egressing blood stages with host daily rhythms
Source: Commun Biol. 2026 Jul 16;9:1485. doi: 10.1038/s42003-026-10641-x (PMC13745455; doi:10.1038/s42003-026-10641-x)
Supplement: Supplementary file 1 [file 42003_2026_10641_MOESM1_ESM.pdf]

nature portfolio

Corresponding author(s): Petra Schneider

Last updated by author(s): Jun 17, 2026

Reporting Summary

Nature Portfolio wishes to improve the reproducibility of the work that we publish. This form provides structure for consistency and transparency in reporting. For further information on Nature Portfolio policies, see our Editorial Policies and the Editorial Policy Checklist.

Statistics

For all statistical analyses, confirm that the following items are present in the figure legend, table legend, main text, or Methods section.

n/a	Confirmed	
☐	☒	The exact sample size (*n*) for each experimental group/condition, given as a discrete number and unit of measurement
☐	☒	A statement on whether measurements were taken from distinct samples or whether the same sample was measured repeatedly
☐	☒	The statistical test(s) used AND whether they are one- or two-sided<br>*Only common tests should be described solely by name; describe more complex techniques in the Methods section.*
☐	☒	A description of all covariates tested
☐	☒	A description of any assumptions or corrections, such as tests of normality and adjustment for multiple comparisons
☐	☒	A full description of the statistical parameters including central tendency (e.g. means) or other basic estimates (e.g. regression coefficient) AND variation (e.g. standard deviation) or associated estimates of uncertainty (e.g. confidence intervals)
☐	☒	For null hypothesis testing, the test statistic (e.g. *F*, *t*, *r*) with confidence intervals, effect sizes, degrees of freedom and *P* value noted<br>*Give P values as exact values whenever suitable.*
☒	☐	For Bayesian analysis, information on the choice of priors and Markov chain Monte Carlo settings
☒	☐	For hierarchical and complex designs, identification of the appropriate level for tests and full reporting of outcomes
☐	☒	Estimates of effect sizes (e.g. Cohen's *d*, Pearson's *r*), indicating how they were calculated

Our web collection on statistics for biologists contains articles on many of the points above.

Software and code

Policy information about availability of computer code

Data collection	no software was used for data collection, the data all arise from experiments.
Data analysis	Data analysis was performed using R version 4.5.0 (2025-04-11). The statistical analyses and packages used are all standard and described in the methods. The include linear models, generalised linear mixed effects models using the glmmTMB package, and Model assumptions were tested using “Dharma” package simulations. Model selection was performed via model fit (AICc), or log-likelihood ratio test (LRT) for nested models. These are all standard procedures available in R.

For manuscripts utilizing custom algorithms or software that are central to the research but not yet described in published literature, software must be made available to editors and reviewers. We strongly encourage code deposition in a community repository (e.g. GitHub). See the Nature Portfolio guidelines for submitting code & software for further information.

Data

Policy information about availability of data

All manuscripts must include a data availability statement. This statement should provide the following information, where applicable:

- Accession codes, unique identifiers, or web links for publicly available datasets
- A description of any restrictions on data availability
- For clinical datasets or third party data, please ensure that the statement adheres to our policy

The datasets supporting the conclusions of this article are available in the Edinburgh DataShare repository: https://doi.org/10.7488/ds/8049

Research involving human participants, their data, or biological material

Policy information about studies with human participants or human data. See also policy information about sex, gender (identity/presentation), and sexual orientation and race, ethnicity and racism.

Reporting on sex and gender	NA
Reporting on race, ethnicity, or other socially relevant groupings	NA
Population characteristics	NA
Recruitment	NA
Ethics oversight	NA

Note that full information on the approval of the study protocol must also be provided in the manuscript.

Field-specific reporting

Please select the one below that is the best fit for your research. If you are not sure, read the appropriate sections before making your selection.

☒ Life sciences ☐ Behavioural & social sciences ☐ Ecological, evolutionary & environmental sciences

For a reference copy of the document with all sections, see nature.com/documents/nr-reporting-summary-flat.pdf

Life sciences study design

All studies must disclose on these points even when the disclosure is negative.

Sample size	determined based on expected effect sizes and ethical considerations for animal use
Data exclusions	no data were excluded from analyses
Replication	Biological replicates were used to confirm outcomes and variation.
Randomization	Allocation was random
Blinding	Blinding was not appropriate during the experiment, as differences in light and food schedules immediately reveal groups. Laboratory data were obtained using a numbering system for individual animals.

Reporting for specific materials, systems and methods

We require information from authors about some types of materials, experimental systems and methods used in many studies. Here, indicate whether each material, system or method listed is relevant to your study. If you are not sure if a list item applies to your research, read the appropriate section before selecting a response.

Materials & experimental systems

n/a	Involved in the study	
☒	☐	Antibodies
☒	☐	Eukaryotic cell lines
☒	☐	Palaeontology and archaeology
☐	☒	Animals and other organisms
☒	☐	Clinical data
☒	☐	Dual use research of concern
☒	☐	Plants

Methods

n/a	Involved in the study	
☒	☐	ChIP-seq
☒	☐	Flow cytometry
☒	☐	MRI-based neuroimaging

Animals and other research organisms

Policy information about studies involving animals; ARRIVE guidelines recommended for reporting animal research, and Sex and Gender in Research

Laboratory animals	Mouse, wildtype (WT) C57BL/6, or Per1-/-;Per2-/- double KO mice (KO) mice
Wild animals	NA
Reporting on sex	All mice were mixed sexes, All mosquitoes were female (males do not feed on blood, nor transmit malaria)
Field-collected samples	NA
Ethics oversight	Experimental procedures complied with all relevant ethical regulations for animal use, specifically the UK Animals Scientific Procedures Act 1986 (SI 2012/3039; PPL PP8390310). The study was conducted in accordance with the local legislation and institutional requirements and was approved by the Animal Welfare and Ethical Review Body at the University of Edinburgh.

Note that full information on the approval of the study protocol must also be provided in the manuscript.

Plants

Seed stocks	NA
Novel plant genotypes	NA
Authentication	NA
